# The impact of pregnancy on women’s oral health-related quality of life: a qualitative investigation

**DOI:** 10.1186/s12903-020-01290-5

**Published:** 2020-10-27

**Authors:** Omid Fakheran, Mahmoud Keyvanara, Zahra Saied-Moallemi, Abbasali Khademi

**Affiliations:** 1 Department of Oral Health and Community Dentistry, Dental Implant Research Center, Dental Research Institute, Isfahan University of Medical Sciences, Isfahan, Iran; 2 Department of Health Services Management, Social Determinants of Health Research Center, Isfahan University of Medical Sciences, Isfahan, Iran; 3 Department of Oral Health and Community Dentistry, School of Dentistry, Isfahan University of Medical Sciences, Isfahan, Iran; 4 Dental Research Center, Department of Endodontics, Dental Research Institute, Faculty of Dentistry, Isfahan University of Medical Sciences, Isfahan, Iran

**Keywords:** Pregnancy, Oral health, Health related quality of life, Qualitative research

## Abstract

**Background:**

Complex psychological and physiological changes occur in women’s body during pregnancy. These changes affect both oral health status and oral health-related quality of life (OHRQoL). In almost all of the previous cross-sectional design studies on pregnant women, generic OHRQoL instruments have been used to measure OHRQoL. While such instruments may be reliable, they may not be appropriate to evaluate the OHRQoL in special populations like pregnant women. The purpose of this study was to investigate the self-perceived factors affecting the OHRQoL among pregnant women.

**Methods:**

In this qualitative descriptive study, twenty- seven pregnant women were recruited from four healthcare centers located in Isfahan city, Iran. The interpretative phenomenological analysis was used to collect and analyze the data. Four criteria of credibility, dependability, transferability, and confirmability were implemented through established procedures to confirm the study rigor.

**Results:**

Three major themes and six sub-themes capturing the impacts of pregnancy on women’s OHRQoL were identified. They covered all areas of life, including daily life, psychological well-being, social life, physical impact, and also barriers to utilization of dental care services. Some new domains such as “dentists’ refusal to treat pregnant women”, “negative feelings about pregnancy” and “concerns about fetal health” were found as important factors which could influence the OHRQoL during pregnancy.

**Conclusion:**

The findings help to better understand the oral health issues impacting women during pregnancy and to achieve person-centered care and improved oral health outcomes in pregnant women. The conceptual framework created based on the results of this study may help health care workers and policy makers for improving the health of pregnant women.

## Introduction

Oral health-related quality of life (OHRQoL) describes a person’s perception of how oral health influences an individual’s quality of life and overall well-being. Locker et al. defined OHRQoL as “the impact of oral disorders on aspects of everyday life that are important to patients and persons, with those impacts being of sufficient magnitude, whether in terms of severity, frequency or duration, to affect an individual’s perception of their life overall” [1]. There are several oral diseases and systemic conditions which may cause functional limitations, psychological discomfort, or social disability and may consequently affect the OHRQoL of the individuals [2–5].

Pregnancy is a unique period of life during a woman’s life. During pregnancy many complex physiologic changes occur in the women’s body, which can adversely affect oral health [6]. Many studies have reported that oral health care needs of pregnant women are completely different from the general population [7–9]. Periodontal disease, Xerostomia, halitosis, and tooth mobility are the most common problems related to oral health during pregnancy [10, 11]. In this regard, some studies have reported that the increasing levels of oral disease have a negative impact on the OHRQoL and perceptions of well-being among pregnant women [12–15]. In a study conducted among 150 pregnant women and 150 non-pregnant women in India, the authors found that the OHRQoL was significantly poorer in pregnant women than in non-pregnant women [13]. These investigators reported that the impact of pregnancy on the OHRQoL was significant in terms of causing psychological discomfort, functional limitation, psychological disability, physical pain, and handicap [13].

In this regard, multiple studies measuring OHRQoL in pregnant and postpartum populations use generic instruments [16]. Generic OHRQoL questionnaires such as oral Health Impact profile (OHIP) and Oral Impacts on Daily Performances (OIDP) are broad measurement scales that measure the OHRQoL in the general population [17, 18].

A recent systematic review reported that the most affected domains of OHRQoL in the general population were different from the most affected domains in pregnant women [16]. Based on this study, the most affected domains of OHRQoL in pregnant women were related to the mental and psychological discomfort [16]; however, the most affected domain in the general population was physical health [2]. Moreover, the psychosocial domains were less affected in the general population [2]. Based on these discrepancies in the results of OHRQoL measurement in the general population vs. pregnant women, generic scales for measurement of OHRQoL may not reflect the actual perception of pregnant women regarding their oral health status and related problems. Previously, it has been documented that condition-specific instruments for assessing OHRQoL may be advantageous over generic measures in various diseases and conditions [19–22]. Specific measures have been developed for specific conditions (e.g., malocclusion) to tap the symptoms and impacts associated with those conditions, which may increase their sensitivity compared with that of generic instruments [19, 23]. Furthermore, another advantage of condition-specific instruments over generic tools is that generic instruments generally have higher “floor effects” (i.e. no impact) since many of the symptoms tapped may not be prevalent or relevant among pregnant women [23]. On the other hand, the generic OHRQoL instruments may not sufficiently reflect some important domains such as oral health-related psychosocial issues in pregnant women [16]. In this regard, it seems to be beneficial to develop a specific OHRQoL measure for pregnant women.

Guyatt et al. described the steps that should be followed during the development of a condition-specific HRQoL measure [24]. This method has also been used successfully for the development of a condition-specific OHRQoL instrument [19]. The first step in this regard is identifying the issues that are important to the individuals suffering from a specific disease or condition [24].

The best scientific approach for collecting the opinions of individuals about their experiences is qualitative research [25, 26]. All the available data in the literature about measuring the OHRQoL during pregnancy are quantitative in nature. Therefore, we believe further research is required to explore, describe, and clarify the pregnant women’s OHRQoL from a qualitative perspective. A qualitative approach allows us to better understand those aspects of oral health disturbances in pregnant women which may have been ignored by quantitative research [27].

In this study, qualitative research methods were used to characterize and describe the women’s experiences about OHRQoL during pregnancy. The aim of this study was exploring and classifying the self-perceived factors behind pregnant women’s OHRQoL, The findings of this study may improve the knowledge of dental health care professionals regarding the issues which may influence the OHRQoL of women during pregnancy. Moreover, these findings may encourage the researchers for conducting more longitudinal studies in the way of need-assessment for developing a pregnancy specific OHRQoL measure.

## Methods

The project was initiated by the first author (OF) as a part of his Ph.D. dissertation, supervised by the other three authors (MK, ZS, and AK). The study group was set up with a researcher in public health dentistry, a professor in social medicine, a professor expert in clinical dentistry, and a researcher expert in the OHRQoL issues. This study group was deliberately fitted in the conceptual framework defined by the World Health Organization [28].

During the first series of meetings, we specified the conditions of feasibility of our project, defined our target population, and started to draw up a preliminary list of the aspects of the OHRQoL during pregnancy. We also conducted a systematic review project to collect all available data regarding the OHRQoL in pregnant women [16].

### Researcher’s subjectivity

This investigation is based on the qualitative approach and the investigators perceive the importance of transparency and reflexivity regarding the researcher’s subjectivity [29]. The first author is a clinician-scientist with 6 years of clinical experience. He has prior experience in the field of oral health issues of pregnant women. The second author (MK) is a professor in medical sociology with 20 years of experience in qualitative studies. The third author (ZS) is an associate professor in the department of community dentistry and is expert in the field of oral health-related quality of life. The fourth author (AK) is a professor of clinical endodontics with 30 years of research experience. The second author (MK) was considered a critical auditor and reviewed the trustworthiness of the qualitative data in the final stages of the study. All authors share an interest in patient-centered perspectives and health-related qualitative research.

### Design

In this qualitative descriptive study, the interpretative phenomenological analysis (IPA) approach was used to collect and analyze the data. The study protocol was approved by the Ethics Committee of Isfahan University of Medical Sciences (ID: 396,722). The purpose and protocol of the study was explained to each participant, and written consent was obtained from the participant before each interview. All the participants were informed that interviews would be audio-recorded. All pregnant women were assured of the confidentiality of the data and the anonymity of the participants. They were also aware that they had the right to leave the session at any time during the study.

### Entrée/setting

This study was conducted in four healthcare centers located in Isfahan city, Iran. To establish rapport and build trust with the pregnant women, the main investigator [OF] assisted the local dentist in the health care center with routine oral health examinations prior to initiating data collection. The interviews were conducted in a quiet meeting room in the healthcare centers on the scheduled day of pregnant women’s dental examinations.

### Sample

Pregnant women of any gestational age attending the healthcare centers, located in Isfahan, Iran, were recruited if they had no history of pregnancy -related complications or chronic health conditions. The eligible women were referred by midwives in the healthcare centers to the research team members. All participants were female, pregnant, 18–45 years old, and able to read and speak Farsi. Purposive sampling method was applied and continued until data saturation. During the recruitment of pregnant women, maximum variations regarding socio-economic aspects, number of pregnancies, and women’s gestational age were considered. The participants completed a demographic and background form prior to the interviews. Data saturation was achieved through twenty-seven interviews, which is in line with other authors reporting data saturation after thirty interviews to develop a specific OHRQoL measure [19]. The mean age of the participants was 27.7 years (SD = 1.23). Descriptive statistics reflecting the participants’ demographic characteristics are given in Table 1.

**Table 1:** key characteristics and self-rated oral health of participants

Woman	Age range	Pregnancy trimester	Highest educational level achieved	Job Status	Gravida status	self-rated oral health
1	21–25	2nd trimester	University Degree	Employed	Primigravida	Fair
2	26–30	2nd trimester	Diploma	Employed	Multigravida	Fair
3	21–25	2nd trimester	Diploma	Housewife	Primigravida	Good
4	31–35	3rd trimester	University Degree	Employed	Multigravida	Excellent
5	26–30	1st trimester	Diploma	Housewife	Primigravida	Good
6	21–25	1st trimester	Diploma	Housewife	Primigravida	Good
7	21–25	3rd trimester	Upper secondary	Housewife	Primigravida	Poor
8	21–25	2nd trimester	Diploma	Housewife	Primigravida	Good
9	26–30	1st trimester	Diploma	Housewife	Primigravida	Poor
10	21–25	2nd trimester	University Degree	Housewife	Primigravida	Good
11	31–35	3rd trimester	Diploma	Employed	Multigravida	Fair
12	26–30	3rd trimester	University Degree	Employed	Multigravida	Fair
13	31–35	1st trimester	Diploma	Housewife	Multigravida	Poor
14	18–20	3rd trimester	Upper secondary	Housewife	Primigravida	Good
15	36–40	3rd trimester	University Degree	Employed	Multigravida	Fair
16	21–25	2nd trimester	University Degree	Employed	Primigravida	Excellent
17	36–40	1st trimester	Diploma	Employed	Multigravida	Good
18	18–20	2nd trimester	Upper secondary	Housewife	Primigravida	Poor
19	21–25	2nd trimester	Upper secondary	Housewife	Multigravida	Good
20	21–25	2nd trimester	Upper secondary	Housewife	Primigravida	Fair
21	36–40	1st trimester	University Degree	Employed	Multigravida	Excellent
22	18–20	3rd trimester	Upper secondary	Housewife	Primigravida	Good
23	36–40	3rd trimester	Diploma	Employed	Multigravida	Poor
24	31–35	1st trimester	Diploma	Employed	Multigravida	Good
25	26–30	3rd trimester	University Degree	Housewife	Primigravida	Poor
26	26–30	2nd trimester	Diploma	Housewife	Primigravida	Fair
27	36–40	2nd trimester	Diploma	Housewife	Multigravida	Fair

### Procedure

The interviews were performed in a quiet room and lasted about 30–40 min. Data were collected using semi-structured, audio-recorded, face-to-face interviews. An interview guide was developed with two open ended requests, “Please, tell me about your oral and dental health condition during your pregnancy” and, “please tell me about the impacts of your oral health condition on your life”. To get a deeper understanding and to clarify some parts of the interview, follow-up questions such as “Can you tell me more about that?” or “Can you clarify that?” were asked. Close to the end of the interview, the participant was asked: “How would you describe the overall condition of your teeth or gums?” A poor-to-excellent response scale was used for assessing self-rated oral health condition of participants. The interviewer’s field notes and memos as well as the participants’ actions were supplied a complementary non-verbal source of data. In this regard we looked around and described what is happening in the setting. We tried to discover what women were doing and saying (for example, speech patterns, facial expressions, gestures, etc.).

We assigned a number to each script and changed each participant’s name to a pseudonym with the aim of maintaining complete confidentiality. Only the first author [OF] had access to the women’s actual names, addresses, and phone numbers.

### Data analysis

All audio-recorded interviews, memos, and field notes were transcribed verbatim and checked for accuracy. Data were analyzed using the interpretative phenomenological analysis (IPA) approach [30, 31]. The IPA was undertaken due to its emphasis on understanding a phenomenon as it is experienced and given meaning in the lived experience of participants [32]. Based on this approach, we were not seeking to confirm the prior themes but we allowed the themes to emerge organically. The analysis process began with listening and iterative cycle of reading interviews a number of times by the primary investigator to ensure that a general sense of story was acquired. In the next steps, the codes were applied to the data and the emergent themes were initially noted. The analysis was managed in MAXQDA software (version 10). As the analysis continued, the earlier transcripts were reviewed in the light of the findings of the later transcripts. Then, connections between the preliminary themes were found for each participant and superordinate themes were identified. The final stage involved naming the themes and translating them into a narrative account [31].

### Rigor enhancement

Four criteria of credibility, dependability, transferability, and confirmability were implemented through established procedures to confirm the study rigor. Prolonged engagement with the pregnant women participating in this study, considering enough time for immersing in the data, as well as checking the findings with the participants (member check) and 3 coauthors (peer debriefing) were established to improve the credibility of the study. In this regard, using maximum variation sampling strengthened the credibility and confirmability of the findings. With the aim of ensuring the transferability of data, results were checked by five pregnant women who had not taken part in the research.

## Results

### Participant characteristics

Twenty-seven pregnant women with an age range of 17–41 years were interviewed. Twenty-two interviews were conducted in the healthcare centers and five interviews were carried out in the participants’ residence. Eight interviews were conducted in the presence of close relatives upon the request of participants. The mean duration of the interview was 47 min (range: 34–58). The socio-demographic and obstetric profiles of the participants are shown in Table 1.

### Findings

From the diverse experiences of participants, we identified three main aspects affecting the OHRQoL during pregnancy: present issues affecting the OHRQoL during pregnancy, regrets affecting the OHRQoL during pregnancy, and future concerns affecting the OHRQoL during pregnancy.

The present issues affecting the OHRQoL during pregnancy included soft tissue problems and gingival bleeding, dental pain and discomfort, disturbance in doing the daily activities, psychological disorders disrupting interactions and causing social disability, and barriers to utilizing dental care services.

Regrets affecting the OHRQoL of pregnant women included regrets about not doing dental checkups and not learning oral health knowledge before pregnancy. And finally, future concerns affecting the OHRQoL during pregnancy consisted of concerns about fetal health and concerns about postponing dental treatment.

**Fig. 1:**
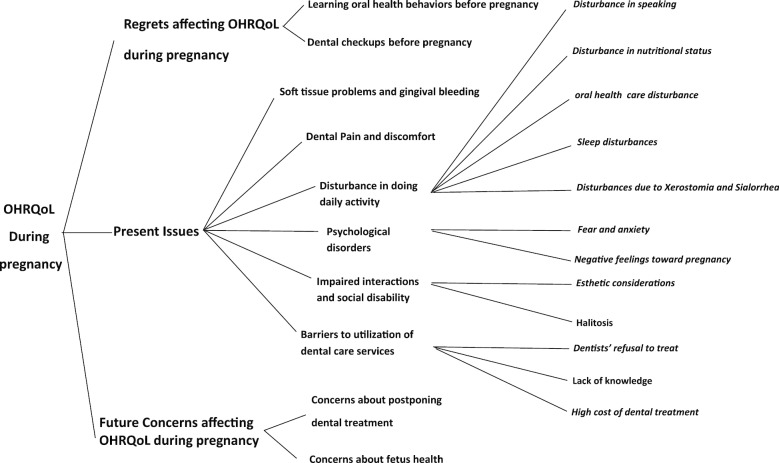
A Conceptual frame work of factors affecting OHRQoL in pregnant women

Figure 1 shows a summary of various factors affecting OHRQoL during pregnancy based on the results of this study.

### Present Issues affecting OHRQoL during pregnancy

#### Soft tissue problems and gingival bleeding

Gingival Inflammation and bleeding were one of the most important problems declared by the women. The occurrence of pregnancy tumors was another issue in this regard. Finding blood in oral cavity makes the women nervous and prevents them from doing routine oral hygiene.

I used to brush my teeth regularly before pregnancy and had no problems, but my gums got swollen after pregnancy, especially in the last months of pregnancy, and started bleeding as soon as they were in contact with a toothbrush. Sometimes when I got up in the morning, I saw blood in my mouth and my pillow was stained with blood.

(P7, 3rd trimester).

#### Dental Pain and discomfort

Acute pulpal pain was one the worst experiences of women during pregnancy. Almost all of the women, who experienced dental pain, didn’t seek any treatment or consume enough drugs to keep their unborn baby healthy. Existence of toothache and the sense of inability to controlling pain was a unique terrible experience reported by the participants.

My tooth was abscessed and my cheek got swollen. I didn’t take painkillers to avoid fetal damage due to drugs. I just tolerated the pain and suffered. My toothache continued for several nights in a row. I just liked someone could extract my tooth, but nobody accepted my treatment.

(P23, 3rd trimester).

#### Disturbance in doing daily activity

All participants described how oral health problems resulted in increasing restrictions on daily activities, both within their professional environment and the household. These restrictions not only affected the participants’ life, but also affected the lives of their relatives and co-workers.

When I feel pain in my gums or teeth, I cannot do my housework. In such conditions, I can’t even stand my children and can’t handle their tasks. Toothache makes me impatient and I can’t do anything.

(P18, 2nd trimester).

The Disturbances classified to five sub-themes including disturbance in speaking, disturbance in doing oral hygiene behaviors, disturbance in nutritional status, sleep disturbance and disturbances related to Xerostomia or Ptyalism.

##### Disturbance in speaking

Speaking is one of the critical skills in daily life. Any pain or problem in oral cavity can lead to speaking restriction. Some participants emphasized the speaking impairment during pregnancy due to dental pain or gingival bleeding.

When I have a toothache, I put my hands on my face and can’t speak until my pain is relieved.

(P25, 3rd trimester).

##### Disturbance in doing oral hygiene behaviors

Based on the women’s narrations, the practice of oral self-care was largely influenced by pregnancy consequences. Gingival bleeding during brushing the teeth, nausea and vomiting of pregnancy and feeling pain in gums after flossing were the most common obstacles of routine oral self-care.

When I put the toothbrush in my mouth, I feel nauseous quickly. For this reason, I rarely brush my teeth. Further, as soon as I floss my teeth, my gums start bleeding, so I don’t use dental floss a lot.

(P5, 1st trimester).

##### Disturbance in nutritional status

The alteration in taste sensation during pregnancy deeply influenced the nutritional behaviors of participated women. The changes in senses of smell and taste were very varied among the participants, but anyway they did occur in all of them. Moreover, based on the narrations, the oral disease symptoms such as gingival bleeding and toothache reduced the chewing ability of some informants.

From the beginning of pregnancy, I feel the taste of foods has changed, so I don’t enjoy most of the foods … Moreover, when I have a toothache, I get very nervous and don’t like to eat anything at all.

(P9, 1st trimester).

##### Sleep disturbances

One of the common problems in almost all of the participants was low sleep quality. Sleep disturbance caused by dental pain and gingival itching was highlighted among the narrations. All of the oral health issues which could influence on the sleep quality or sleep duration of pregnant women were classified in this section.

I can’t sleep at all during the nights I have a toothache. I can’t take strong drugs because they are harmful to the child. For this reason, I don’t have energy and can’t do my works.

(P15, 3rd trimester).

##### Disturbances due to Xerostomia and Sialorrhea

Saliva disorders were common among the participants. These disorders were very different among the interviewed women. In some cases the reduction in saliva secretion and its related outcomes such as disturbance in chewing food and speaking were reported. However some others talked about the increased salivation which leads to nausea and vomiting, especially in the first trimester of pregnancy. Interestingly, the participants considered these problems as normal consequences of pregnancy and they never sought any treatment in this regard.

My saliva has increased since I got pregnant … At night when I sleep, my saliva flows onto my pillow.

(P16, 2nd trimester).

#### Psychological disorders

The analysis of narrations apparently showed that oral health problems caused several psychological disorders for pregnant women.

These psychological themes, apart from the physical conditions, independently emerged from the narrations.

##### Fear and anxiety

Experience of dental pain during previous or current pregnancy, created some fear and anxiety in the participants. The high acute pain of pulpitis and believing in impossibility of dental treatment during pregnancy were the two main elements of this fear. Visiting the dentists who were apparently reluctant to treat pregnant women was another reason of this phobia among participants.

In my previous pregnancy, I had a toothache, a terrible experience for me. Doctors couldn’t do anything for me. Therefore, I treated my caries teeth before the current pregnancy. However, I am always worried about toothache and the same old problems.

(P4, 3rd trimester).

##### Negative feelings toward pregnancy

The occurrence of untreated oral health problems and remaining in such situation for several days particularly increased the negative feelings about pregnancy in participants. The sense of inability to cope with the problem and considering the growing fetus as the reason of this suffering apparently emerged from the words of pregnant women.

I was very excited during my first pregnancy. I always talked to my child in my belly and loved her a lot. But in the current pregnancy, I have no sense of motherhood because I have had toothache from the beginning of pregnancy. I am always suffering and can’t do anything. I wish it would end sooner so that I can repair my teeth.

(P18, 2nd trimester).

#### Impaired interactions and social disability

The oral health conditions deeply affected the social life of pregnant women and altered their interactions with other people during pregnancy. Two sub-themes emerged in this regard from the narrations are self-perceived halitosis and esthetic consideration.

##### Halitosis

The participants commonly reported some problems about their bad breath. The halitosis in pregnant women might be true halitosis detected by the relatives or the self-perceived halitosis. Both of these disorders significantly decreased the women’s self-esteem and resulted in impaired interactions with other people.

From the very beginning of pregnancy, I smell blood in my mouth. That's why when I want to talk to someone, I try not to get too close to him/her.

(P10, 2nd trimester).

##### Esthetic considerations

The oral soft tissue appearance, specially the swallowing and alteration of the color in anterior gingiva were one of the important concerns among the pregnant women.

Since my gums are red and swollen, I think it is disgusting for others. I never want to go out or see someone. I try not to laugh to hide my gums. I always put my hands in front of my mouth and talk (she laughs and unconsciously puts her hands in front of her mouth).

(P20, 2nd trimester).

#### Barriers to utilization of dental care services

The importance of accessing to dental care services and barriers to utilization of professional dental treatments strongly emerged from the narrations. Based on the analysis of data the obstacles of delivering dental treatments to pregnant women summarized in three sub-themes. All of these concepts could efficiently affect the OHRQoL of participants during pregnancy.

##### Lack of knowledge and believing myths about professional dental care

Unsupported misconceptions about professional dental treatments during pregnancy were common among the participants in this sample. Participants believed that routine dental procedures would harm them or their baby. The origin of such misconception was rooted in the beliefs of their husbands and elder family members.

When I got gingival bleeding, my husband didn’t let me go to a dentist. My husband and I think dental treatment harms the baby. In my opinion, anesthetics, dental materials and devices can have negative effects on baby’s health.

(P20, 2nd trimester).

##### High cost of dental treatment

Economic problems of family and the high cost of dental treatments were another obstacles of attending dental offices. Based on the opinions of participants, dental treatment didn’t have high priority compared with other health issues. Moreover, many of them didn’t have any information about the free dental health services which is available in public healthcare centers for pregnant women.

I am very upset that I have decayed teeth. However, I can’t help it because my husband has a low income. The costs of other pregnancy tests and sonography are very high. I may be able to repair my teeth after pregnancy when other costs are reduced.

(P27, 2nd trimester).

##### Dentists’ refusal to treat pregnant women

Another important obstacle of accessing dental care services is the refusal of treatment by dentists during pregnancy. Based on the common experience of women, dentists were obviously reluctant to treat pregnant women.

When my tooth was abscessed, I referred to some dentists but none accepted my treatment. All of them said it is not possible to treat teeth during pregnancy.

(P25, 3rd trimester).

### Regrets affecting OHRQoL during pregnancy

Based on the analyzing of narrations, some concerns of women during their pregnancy were rooted in the past and before pregnancy. These issues were mostly the regrets about the past. The regrets were created based on the hard experiences of dental problems during pregnancy. The two main regrets affecting the OHRQoL were “Dental checkups” and “Learning oral health behaviors” before pregnancy. The women considered these themes as the opportunity which had been lost.

I have done all medical care before pregnancy. The only thing I haven’t done is referring to a dentist to check my teeth, something that has annoyed me a lot.

(P11, 3rd trimester).

Now that I am pregnant and have gingival bleeding and pain, I highly regret why I haven’t learned the preventive methods for these problems before.

(P2, 2nd trimester).

### Future Concerns affecting OHRQoL during pregnancy

Worry about the future problems caused by the current oral cavity problems were another theme which has been extracted from the narrations. These worries could be classified as “Concerns about postponing dental treatment” and “Concerns about fetus health”. The inability of treating teeth during pregnancy made women to think about the consequences of delay in treatment. Women believed that postponing of dental treatment would cause more severe problems and morbidities for themselves. On the other hand, they were seriously concerned about the negative impacts of their oral disease on baby’s health. Almost all of the participants believed that the presence of dental caries and gingival bleeding in mother’s mouth would be harmful for the unborn baby.

… I am worried that my severe toothache will shock the baby…

(P15, 3rd trimester).

It is not possible to treat my teeth during pregnancy. I am worried that I will have more decayed teeth until three months later when my child will be borne! I'm worried that my teeth will fall out during this time!

(P7, 3rd trimester).

## Discussion

This qualitative study was conducted to describe the OHRQoL of women during pregnancy. It sought to examine the subjective experience of pregnant women about their OHRQoL during the gestation period using the phenomenological qualitative approach. Inductive qualitative analysis of lived experiences allowed the discovery of the most important domains of OHRQoL according to pregnant women.

Based on the results of this study, the themes which may affect OHRQoL during pregnancy can be classified in three main categories named as "present issues", "regrets" and "future concerns". Many of these factors previously have been met in classic measures such as OHIP-14 and OIDP. The themes such as dental pain and discomfort, disturbance in doing the daily activities, psychological disorders and social disability are similar with issues included in generic OHRQoL instruments [17, 18]. Some domains of OHRQoL found in this study were specific to pregnant women and might not emerge from the generic scales such as OHIP-14 or OIDP. Domains such as “dentists’ refusal to treat pregnant women”, “negative feelings toward pregnancy”, and “concerns about fetal health” are in line with the results of previous studies related to oral health issues in pregnant women. Another qualitative study conducted in Iran reported the dentists’ avoidance of treating pregnant women [33]. In this report, the dentists’ fear of “legal consequences of potential complaints about their practice” was mentioned as the reason for this issue [33]. In a qualitative study among pregnant adolescent women in the United States of America, one of the main themes was “believing myths and having misconceptions about oral health” [27]. This theme was also reported in another qualitative study in Kuwait [34]. The result of a systematic review also showed that the “myths about dental treatment safety” and “dentists’ unwillingness to treat women during their pregnancy” beside some other factors were the main barriers to professional dental care during pregnancy [35].

The OHRQoL in pregnant women has been previously studied using quantitative methods. All of these studies have used the routine generic questionnaires which have been designed for general populations [13, 36, 37]. Therefore, it is obvious that these studies could not have found any new specific issue related to the quality of life in pregnant women. Almost all of these studies have reported that the oral health status of pregnant women is significantly associated with their OHRQoL scores [13, 36, 37]. It means that the OHRQoL during pregnancy can be negatively influenced by periodontal diseases and dental caries [12, 38, 39]. However, in this phenomenological investigation we sought to find out the subjective factors in this regard. The results of this study clearly showed the perceptions and feelings of pregnant women about their oral health problems. These findings were critical as pregnant women might not be asked to describe their oral health-related emotional and social problems during routine examinations or at busy clinics. For instance, finding the regrets about past events and the future concerns related to the OHRQoL could be obtained only through the interpretative phenomenological approach used in this study. It should be noted that the current generic questionnaires may not cover these aspects of OHRQoL during pregnancy. In order to properly assess the OHRQoL during pregnancy, we should take into account not only the general aspects of the OHRQoL, which is available in generic scales, but also the specific issues emerged from this qualitative study.

The results of this study can also help clinicians to better understand the actual and perceived issues, problems, limitations, restrictions, and adaptation strategies specific to pregnant women. Understanding the factors impacting the pregnant women outside the dental office can help to achieve person-centered care and improved oral health outcomes [40, 41]. Furthermore, data obtained from this study may help future investigations regarding the need-assessment for developing a pregnancy specific OHRQoL measure.

It should be mentioned that clinical dental examinations of women before pregnancy and during the gestation period should not be neglected [38]. All pathologic changes in the oral cavity, including dental caries, periodontal diseases, or pregnancy tumors, can negatively impact the women’s OHRQoL during pregnancy [13, 36, 37]. Hence, the results of qualitative investigations in the field of OHRQoL can be taken only as complementary outcomes beside clinical parameters.

The present study showed the importance of oral health care knowledge and behaviors before pregnancy. The results of cross-sectional studies around the world have also highlighted the lack of oral health knowledge among pregnant women [42–44]. Accordingly, some clinical trials have suggested that proper education methods for women before pregnancy can significantly increase their oral health knowledge and consequently improve their oral health status during pregnancy [45–48].

A limitation of this study was that it was undertaken in four public healthcare centers of a single city where primary oral health care services are free for the pregnant women. Further, the women referring to non-governmental health care centers during their pregnancy might have different restrictions, adaptation strategies, or perceived issues regarding the OHRQoL. The experiences of pregnant women might also be very different in other cultural or socioeconomic environments. It should be noted that many studies have investigated the pregnancy issues and concerns, but few studies have focused on the OHRQoL. Also, there is no qualitative study on the OHRQoL during pregnancy. Hence, in order to better understand the factors affecting the OHRQoL during pregnancy, it is essential to conduct several qualitative studies in different places to confirm the findings of the present study.

## Conclusion

This qualitative study shows specific issues related to the OHRQoL in pregnant women based on their lived experiences. The findings help to better understand the oral health issues impacting women during their pregnancy and to achieve person-centered care and improved oral health outcomes in pregnant women.

## Data Availability

The dataset supporting the conclusions of this article available and will be presented based on request.

## Abbreviation

OHRQoL: Oral health related quality of life

## References

[1] Locker D, Allen F (2007). What do measures of ‘oral health-related quality of life’measure?. Community Dent Oral Epidemiol.

[2] Haag D, Peres K, Balasubramanian M, Brennan D (2017). Oral conditions and health-related quality of life: a systematic review. J Dent Res.

[3] Gerritsen AE, Allen PF, Witter DJ, Bronkhorst EM, Creugers NH (2010). Tooth loss and oral health-related quality of life: a systematic review and meta-analysis. Health Quality Life Outcomes.

[4] Ferreira MC, Dias-Pereira AC, Branco-de-Almeida LS, Martins CC, Paiva SM (2017). Impact of periodontal disease on quality of life: a systematic review. J Periodontal Res.

[5] Cervino G, Terranova A, Briguglio F. Diabetes: oral health related quality of life and oral alterations. 2019, 2019:5907195 10.1155/2019/5907195 PMC6442307 31011577

[6] Hartnett E, Haber J, Krainovich-Miller B, Bella A, Vasilyeva A, Kessler JL (2016). Oral health in pregnancy. J Obstet Gynecol Neonatal Nurs.

[7] Giglio JA, Lanni SM, Laskin DM, Giglio NW (2009). Oral health care for the pregnant patient. J Can Dental Assoc.

[8] Mills LW, Moses DT (2002). Oral health during pregnancy. MCN Am J Maternal/Child Nurs.

[9] Lee A, McWilliams M, Janchar T (1999). Care of the pregnant patient in the dental office. Dent Clin N Am.

[10] Russell SL, Mayberry LJ (2008). Pregnancy and oral health: a review and recommendations to reduce gaps in practice and research. MCN Am J Maternal/Child Nurs.

[11] Hughes D (2010). Oral health during pregnancy and early childhood: barriers to care and how to address them. J Calif Dental Assoc.

[12] Moimaz SA, Rocha NB, Garbin AJ, Garbin CA, Saliba O (2016). Influence of oral health on quality of life in pregnant women. Acta Odontologica Latinoamericana AOL.

[13] Geevarghese A, Baskaradoss JK, Sarma PS (2017). Oral health-related quality of life and periodontal status of pregnant women. Matern Child Health J.

[14] Acharya S, Bhat PV (2009). Oral-health-related quality of life during pregnancy. J Public Health Dent.

[15] Cornejo C, Rossi G, Rama A, Gomez-Gutierrez N, Alvaredo G, Squassi A, Klemonskis G (2013). Oral health status and oral health-related quality of life in pregnant women from socially deprived populations. Acta odontologica latinoamericana : AOL.

[16] Fakheran O, Saied-Moallemi Z, Khademi A, Sahebkar A (2020). Oral health-related quality of life during pregnancy: a systematic review. Curr Pharm Des.

[17] Slade GD, Spencer AJ (1994). Development and evaluation of the Oral Health Impact Profile. Community Dent Health.

[18] Adulyanon S, Sheiham A, Slade G (1997). Measuring oral health and quality of life.

[19] Benson PE, Cunningham SJ, Shah N, Gilchrist F, Baker SR, Hodges SJ, Marshman Z (2016). Development of the Malocclusion Impact Questionnaire (MIQ) to measure the oral health-related quality of life of young people with malocclusion: part 2–cross-sectional validation. J Orthod.

[20] Gondivkar SM, Bhowate RR, Gadbail AR, Gondivkar RS, Sarode SC, Sarode GS, Patil S (2018). Impact of oral submucous fibrosis on oral health-related quality of life: a condition-specific OHRQ oL-OSF instrument analysis. Oral Dis.

[21] Gondivkar SM, Bhowate RR, Gadbail AR, Gondivkar RS, Sarode SC, Saode GS (2019). Comparison of generic and condition-specific oral health-related quality of life instruments in patients with oral submucous fibrosis. Qual Life Res.

[22] He S, Wang J, Wei S, Ji P (2017). Development and validation of a condition-specific measure for chronic periodontitis: Oral health impact profile for chronic periodontitis. J Clin Periodontol.

[23] Sischo L, Broder H (2011). Oral health-related quality of life: what, why, how, and future implications. J Dent Res.

[24] Guyatt GH, Bombardier C, Tugwell PX (1986). Measuring disease-specific quality of life in clinical trials. CMAJ.

[25] IPA O. Exploring lived experience. Psychologist 2005;18(1):20.

[26] Smith JA (1996). Beyond the divide between cognition and discourse: using interpretative phenomenological analysis in health psychology. Psychol Health.

[27] Murphey C (2013). Oral health experiences of pregnant and parenting adolescent women: a qualitative descriptive study. Int J Nurs Stud.

[28] Orley J, Kuyken W, The WHOQOL Group (1994). The development of the WHOQOL. Quality of life assessment: International perspectives.

[29] Mauthner NS, Doucet A (2003). Reflexive accounts and accounts of reflexivity in qualitative data analysis. Sociology.

[30] Biggerstaff D, Thompson AR (2008). Interpretative phenomenological analysis (IPA): A qualitative methodology of choice in healthcare research. Qual. Res. Psychol..

[31] Smith JA, Shinebourne P (2012). Interpretative phenomenological analysis.

[32] Smith JA, Osborn M (2015). Interpretative phenomenological analysis as a useful methodology for research on the lived experience of pain. Br J Pain.

[33] Ghorbani Z, Pakkhesal M, Arshi S, Eghbal MJ, Deghatipour M, Tennant M, Ardakani HM (2017). Challenges impeding integration of oral health into primary health care. East Mediterr Health J.

[34] Al Khamis S, Asimakopoulou K, Newton J, Daly B (2016). Oral health knowledge, attitudes, and perceptions of pregnant Kuwaiti women: a qualitative study. JDR Clin Transl Res.

[35] Rocha JS, Arima L, Chibinski AC, Werneck RI, Moysés SJ, Baldani MH (2018). Barriers and facilitators to dental care during pregnancy: a systematic review and meta-synthesis of qualitative studies. Cadernos de saude publica.

[36] Lu HX, Xu W, Wong MC, Wei TY, Feng XP (2015). Impact of periodontal conditions on the quality of life of pregnant women: a cross-sectional study. Health Quality Life Outcomes.

[37] Acharya S (2008). Oral health-related quality of life and its associated factors in an Indian adult population. Oral Health Prev Dent.

[38] Martinez-Beneyto Y, Montero-Martin J, Garcia-Navas F, Vicente-Hernandez A, Ortiz-Ruiz AJ, Camacho-Alonso F (2019). Influence of a preventive program on the oral health-related quality of life (OHRQoL) of European pregnant women: a cohort study. Odontology.

[39] Musskopf ML, Milanesi FC, Rocha JMD, Fiorini T, Moreira CHC, Susin C, Rosing CK, Weidlich P, Oppermann RV (2018). Oral health related quality of life among pregnant women: a randomized controlled trial. Braz Res.

[40] Bedos C, Loignon C (2011). Patient-centred approaches: new models for new challenges. J (Can Dental Assoc).

[41] Lee H, Chalmers NI, Brow A, Boynes S, Monopoli M, Doherty M, Croom O, Engineer L (2018). Person-centered care model in dentistry. BMC Oral Health.

[42] Gonik B, Wilson E, Mayberry M, Joarder BY (2017). Pregnant patient knowledge and behavior regarding perinatal oral health. Am J Perinatol.

[43] Lubon AJ, Erchick DJ, Khatry SK, LeClerq SC, Agrawal NK, Reynolds MA, Katz J, Mullany LC (2018). Oral health knowledge, behavior, and care seeking among pregnant and recently-delivered women in rural Nepal: a qualitative study. BMC Oral Health.

[44] Penmetsa GS, Meghana K, Bhavana P, Venkatalakshmi M, Bypalli V, Lakshmi B (2018). Awareness, attitude and knowledge regarding oral health among pregnant women: a comparative study. Niger Med J J Nigeria Med Assoc.

[45] Jeihooni AK, Jamshidi H, Kashfi SM, Avand A, Khiyali Z (2017). The effect of health education program based on health belief model on oral health behaviors in pregnant women of Fasa City, Fars Province, South of Iran. J Int Soc Prev Community Dent.

[46] Vamos CA, Thompson EL, Avendano M, Daley EM, Quinonez RB, Boggess K (2015). Oral health promotion interventions during pregnancy: a systematic review. Commun Dent Oral Epidemiol.

[47] Deshpande AN, Dhillon SJ, Somanna KS, Dave BH, Porwal PA, Macwan CS (2015). Impact of perinatal oral health care education programme on the knowledge, attitude and practice behavior amongst gynaecologists of Vadodara City. J Indian Soc Pedodont Prev Dent.

[48] Al Khamis S, Asimakopoulou K, Newton T, Daly B (2017). The effect of dental health education on pregnant women's adherence with toothbrushing and flossing—a randomized control trial. Commun Dent Oral Epidemiol.

